# History of a Case of Remarkable Coloured Secretion from the Skin

**Published:** 1847-01

**Authors:** C. D. Purdon

**Affiliations:** Belfast


*History of a case of remarkable coloured Secretion from the Skin.* By C. D. PURDON, M. D., Belfast.—Barbara Murphy, æt. 40, an inmate of the Infirmary attached to the Belfast Charitable Society, the mother of two children: catamenia regular up to the last six months; attributes the first commencement of her state to a fever with which she was attacked about twenty years ago, immediately after which she experienced a pain in the ball of the great toe of the right foot, terminating in a swelling of the same part. Both ankles became painful and swollen; a short time after this ascites supervened; all these symptoms subsided on her becoming pregnant, during which time she enjoyed perfect health. About three weeks after her confinement she was seized with pain, accompanied with swelling, in the joint of the first finger of the right hand, which gradually attacked each joint of the upper extremities in succession, and spread thence to the lower, commencing above. After some time the wrists, ankles, and smaller joints of the hands, became distorted and nodose, in which state they have since continued. From this time nothing remarkable was to be observed in her state, but she continued to suffer from occasional attacks of rheumatism, and was almost always confined to bed, until three years ago, when, during an attack of rheumatic fever, the heart for the first time became affected, after which anasarca and hydrothorax supervened. These were partly relieved by a severe diarrhœa, but on its subsidence both became greatly aggravated; however, they were not only kept in check but much ameliorated by the different remedies employed. Some months ago they returned with such severity as to threaten a sudden termination of her life; when at the worst a miliary eruption appeared on the trunk, greatest in the epigastric region, from which a clear serous discharge flowed in such quantities as literally to wet the bed; there was also a great moisture on the legs, which had blisters on them, in place of the eruption; this of course was attended with the greatest relief, and the breathing became almost free. The discharge continued for some days, after which it ceased, and symptoms of dyspnœa returned with great severity for fourteen days, when, after having a sense of prickling over the whole body for about twelve hours, the eruption again appeared, attended with the discharge, and causing the same relief. In this state of alternate relapse and recovery she has been for the last two months, the duration of the paroxysms being either eight or fourteen days; but the most curious point in the case is, that the serous discharge has changed very much in its character for the last four or five attacks, being nearly alternately blue and straw-coloured, or yellow, almost like pure bile. When the blue discharge appears she is aware of its advent by a mouldy smell and a prickly sensation, which precedes it invariably for twelve hours; the yellow is not attended by either of these. The blue always appears along the posterior part of the chest; the yellow generally proceeds from the abdomen and back of the neck, and rarely from the back: the blue never has appeared on the abdomen; the two colours have been procured from the different parts at the same time. The discharge from the extremities has never been coloured. In place of catamenia there is a discharge of a reddish green colour. As to treatment every remedy has been tried without relief to any of the symptoms, either of the rheumatic or cardiac affections The yellow colour is tolerably permanent, the blue, however, fades; she has not taken any preparation of iodine for some years, and at present uses only opiates and saline draughts. In addition, it is worthy of remark, that a very peculiar elongation of the *quick* under each great toe nail has taken place. This became manifest on the nail being paired, and now appears like a loose fold of flesh, which hangs over the ball of the toe, and resembles in shape the bony nail.—*Dublin Quarterly Journal of Medical Science.*

